# Manifestation of pityriasis rosea and pityriasis rosea‐like eruptions after Covid‐19 vaccine: A systematic review

**DOI:** 10.1002/iid3.804

**Published:** 2023-04-12

**Authors:** Iman Khan, Ahmed A. Elsanousi, Amena M. Shareef, Sameer S. Tebha, Aabiya Arif, Sana Gul

**Affiliations:** ^1^ Department of Medicine Ziauddin Medical University Karachi Sindh Pakistan; ^2^ Department of Medicine University of Medical Science and Technology Khartoum Sudan; ^3^ Department of Medicine Deccan College of Medical Sciences Hyderabad India; ^4^ Department of Neurosurgery and Neurology Jinnah Medical and Dental College Karachi Sindh Pakistan; ^5^ Department of Dermatology Jinnah Medical and Dental College Karachi Sindh Pakistan

**Keywords:** cutaneous side‐effects, infection, papulosquamous, SARS‐CoV‐2 vaccine, skin diseases, vaccine hesitancy

## Abstract

**Background**: After introducing Covid‐19 vaccines, a few side effects were reported, pityriasis rosea being one of them. Therefore, this study will systematically review its manifestation afteradministration.**Methods**: Databases were searched, covering a timeline from December 1, 2019 to February 28, 2022. Data were independently extracted and accessed for bias. SPSS statistical software version 25 was used for appropriate inferential statistics. **Results**: Thirty‐one studies were included for data extraction after screening following the eligibility criteria. A total of 111 people were identified to have developed pityriasis rosea or pityriasis rosea‐like eruptions after vaccination, out of which 36 (55.38%) were female. The average age of incidence was calculated to be 44.92 years, and 63 (62.37%) people presented after administration of the first dose. It was found popularly in the trunk area, either asymptomatically or with mild symptoms. Meantime the onset, was 8.58 days, and meantime it took to recover, was 6.44 weeks. **Conclusion**: The association between pityriasis rosea and pityriasis rosea‐like eruptions after Covid‐19 vaccines was established, but given the scarcity of studies, there is a need to conduct different clinical trials to confirm this association further and study the etiology and mechanism of the disease.

## Graphical abstract

Immune response to SARS‐CoV‐2 mRNA vaccine.

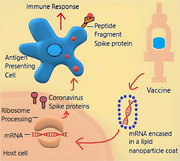

## 1 INTRODUCTION

Covid‐19, caused by severe acute respiratory syndrome coronavirus 2 (SARS‐CoV‐2), was declared a global pandemic by the World Health Organization (WHO).^1^ With the number of infected cases drastically increasing, it posed a serious global threat to our healthcare system due to inundated hospitals and resource deprivation.^2^ Hence, there was an urgent need to take necessary action to control the spread of the infection. Since the first deleterious wave of coronavirus, there have been countless efforts to develop efficacious strategies like drugs and vaccines to abate the risk of catastrophic spread.^3^ Around nine billion total vaccine doses by companies like Pfizer,® Moderna,® and AstraZeneca® were administered on January 19, 2022.^4^ Most of them appeared to be safe and successful in possibly controlling the pandemic.^5^ These vaccines trigger an immune reaction by exposing the weakened antigen to the body and producing antibodies against the infection. After administration, some mild to moderate side effects were reported, which include fever, headache, pain at the injection site, fatigue, chills, diarrhea, and muscle pain.^6^

Cutaneous manifestations are generally common and their incidence was found to be 1%–3% among indoor patients, due to adverse drug reactions, in developed countries.^7^ A study also reported cutaneous reactions to be secondary to COVID‐19 vaccination, with a 3.8% pooled prevalence (95% confidence interval [CI]: 2.7%−5.3%).^8^ These reactions range from exanthema to inflammation, including lichen planus, chilblain‐like lesions, maculopapular or morbilliform rash, erythema multiforme, nonspecific hypersensitivity eruptions, facial dermal filler reactions, reactivation of the varicella‐zoster virus, urticaria, and pityriasis rosea (PR) pityriasis rosea‐like rash (PR‐LE).^9^ Inflammatory skin manifestations have been divided, according to their reaction pattern, into five main categories; vascular, red diffuse eruptions; vesicobullous, related with blisters; dermal, affecting deep component in the skin with little or no epidermal change; eczematous; and papulosquamous, scaly, and red conditions.^10^

PR, an inflammatory papulosquamous disorder, globally accounts for 2% of dermatological cases, other papulosquamous reactions are psoriasiform, annular, lichenoid, and erythroderma. Common morphological presentation for PR, in about 80% of the patients, was of a “herald patch”; 2−10 cm oval, salmon‐colored plaques, known to be like a “Christmas tree” pattern along the cleavage lines.^11^ There is no defined etiology of the disease, but the patch is predominated by T cells and lacks natural killer and B cells, hinting toward a weakened immune system. Possible causes can be bacterial, viral, drug‐induced, or vaccine‐adverse effects. It has known to be firmly associated with the reactivation of human herpesvirus 6 (HHV6) and HHV7. If not treated timely, bacterial superinfections and post‐inflammatory hyperpigmentation may occur. It might also lead to miscarriage in rare circumstances in pregnant women.^12^

Since PR is a rare disease, there is very scarce and scattered literature available on it. Hence it is essential to systematically review it while highlighting its clinical and diagnostic histological features and appropriate treatment options, prompt diagnosis, and treatment. It can also aid in avoiding costly and unnecessary investigations. Furthermore, at devastating times like these, where scientists worldwide are urging people to get vaccinated to combat the SARS‐CoV‐2 virus and impede its viral transmission, it is crucial to study the possible cutaneous side effects of vaccination. Such findings on the degree of awareness can help address this concern against getting vaccinated and counsel people accordingly to reduce vaccine hesitancy. Hence this study is being conducted to study the prevalence of PR, and pityriasis rosea‐like eruptions (PRLE) in individuals who have been vaccinated against Covid‐19.

## 2 MATERIALS AND METHODS

Extensive literature research was performed using the Preferred Reporting Items for Systematic Reviews and Meta‐Analysis (PRISMA) guidelines.^13^ The protocol was registered on PROSPERO (CRD42022313143).

### 2.1 Search methods

**Figure 1:**
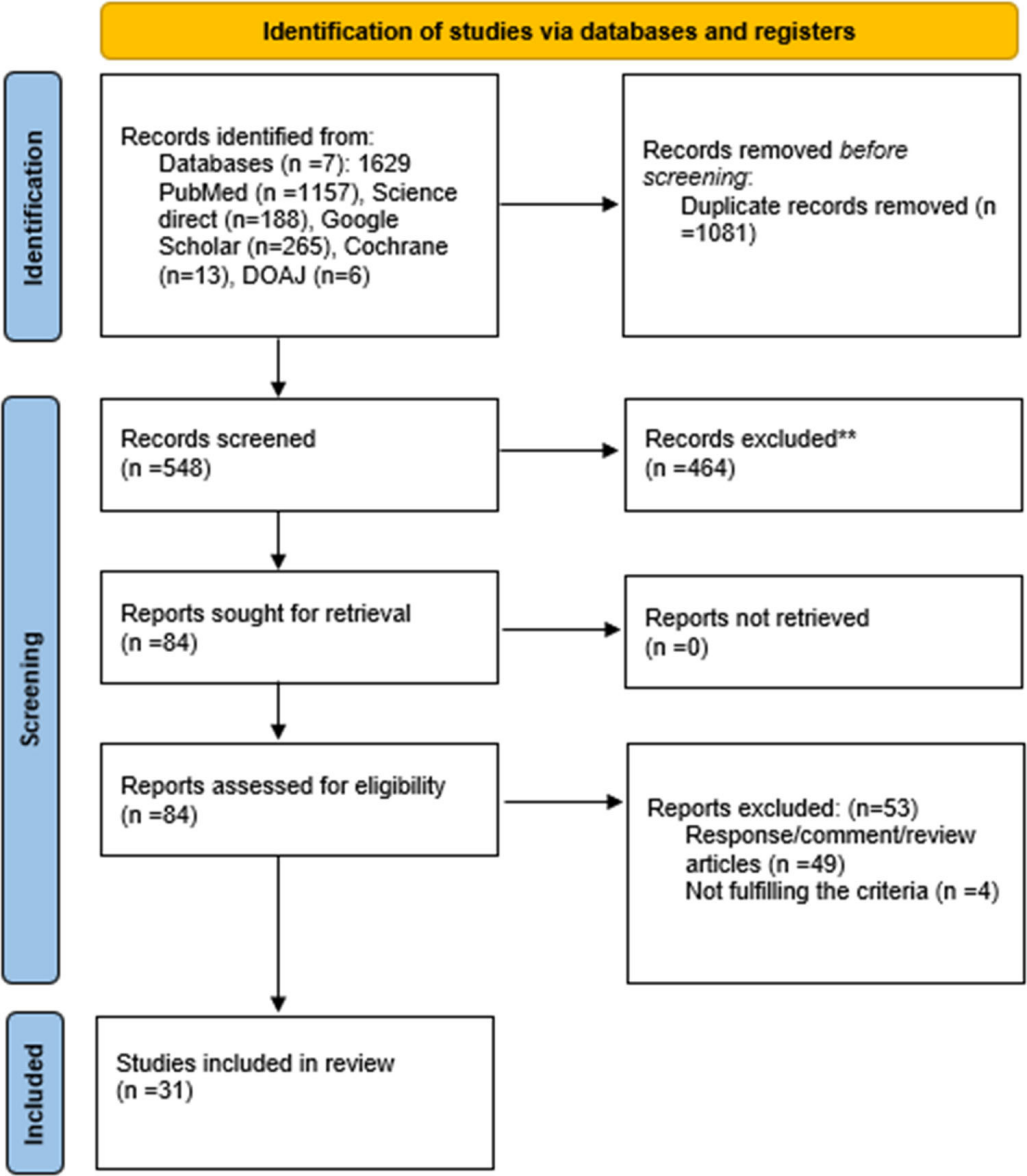
PRISMA flowchart. PRISMA, preferred reporting items for systematic reviews and meta‐analysis.

We implemented the Boolean (and/or) logic using the following set of Mesh terms; “Covid‐19,” “SARS‐CoV‐2,” “vaccination,” “immunization,” “side effects,” “cutaneous reactions, “and “Pityriasis Rosea” over the following authoritative databases: PubMed, Science Direct, Google Scholar, Cochrane Library, and Directory of Open Access Journals. Additionally, MedRxiv and BioRxiv were searched to identify preprinted studies, covering a timeline of December 1, 2019 to February 28, 2022. The search strategy used in each database is provided in Supporting Information: Table S1. Key reference lists were also scanned to find more relevant research. The search was limited to human studies. PRISMA flowchart (Figure 1) was used to record the flow of information through different phases of our study.

### 2.2 Study selection

One thousand six hundred twenty‐nine studies were identified after a literature search, and 1081 of them were excluded due to duplication using Endnote. Two screening stages were conducted by two authors separately; I. K. and A. A., and a third author; S. S. T., resolved any disagreements. In the first stage, the abstracts and titles of the remaining 548 reports were individually screened to filter out studies relevant to our research question, which were 84. Furthermore, in the second stage, any human and observational study was selected, including case series, case reports, cohort, and cross‐sectional studies. Review articles, responses, and commentaries were excluded, 49 in number. After individually screening the full‐text links, 31 studies were finalized that met the eligibility criteria and were used for data extraction and synthesis.^14^, ^15^, ^16^, ^17^, ^18^, ^19^, ^20^, ^21^, ^22^, ^23^, ^24^, ^25^, ^26^, ^27^, ^28^, ^29^, ^30^, ^31^, ^32^, ^33^, ^34^, ^35^, ^36^, ^37^, ^38^, ^39^, ^40^, ^41^, ^42^, ^43^, ^44^

### 2.3 Eligibility criteria

To avoid selection bias, inclusion criteria were set up for the target population to contain participants that: (1) have received at least one dose of the following Covid‐19 vaccination: mRNA (Moderna BNT162b2 and Pfizer BioNTech BNT162b2), Adenovirus vectored (AstraZeneca AZD1222, CanSino Ad5‐nCoV, Gamaleya Sputnik V, Janssen Pharm Ad26.COV.S), Inactivated (BIBP‐CorV Beijing Sinopharm, CoronaVac Sinovac, Inactivated Wuah Sinopharm, BBV152 Bharat Biotech), Recombinant subunit (NVX‐CoV2373 Novarax, ZF2001 Zhei Longcom, SCB‐2019 Clover), and DNA (INO‐4800 Inovio); (2) people diagnosed with PR and PR‐LE in the study irrespective of the diagnostic criteria; (3) are of age 18 or above; (4) male or/and female. Studies were excluded if participants; (1) had a history of PR and PR‐LE before presentation; (2) had been previously or recently tested positive for covid‐19; (3) had any comorbidities or autoimmune/systemic disease; (4) were on any immunosuppressive drug; (5) currently have any other viral disease.

### 2.4 Data extraction and synthesis

Two investigators independently scanned the 31 potential studies. The following data were extracted: Title, author names, study design, year of publication, sample size, age, and gender (male/female) of patients, name, and dose of vaccines administered, distribution, and time of onset of the cutaneous reaction after any dose of vaccination, morphological, its histological and clinical features, treatment prescribed, and days taken to recover. Discrepancies, if any, were settled by a third author.

Data extracted was then organized systematically in a shared excel spreadsheet, categorized according to a standardized data extraction form. SPSS statistical software Version 25, with a *p*‐value of <.05 considered significant and a 95% CI, was used for appropriate inferential statistics. Descriptive data were analyzed and presented as frequency/percentage, and continuous data were reported as mean with standard deviation. Finally, qualitative data were combined and analyzed to conclude the findings.

### 2.5 Risk of bias assessment

Appropriate quality assessment tools for case study/series and observational studies developed by the National Heart, Lung, and Brain Institute and Research Triangle Institute International were used to evaluate internal validity and remove the risk of bias. (13) Two authors independently assessed the quality of selected articles; A. A. E., and A. M. S., as shown in Supporting Information: Tables S2 and S3. Case studies and series were scored out of 7 and categorized as good quality (score 6−7), fair quality (score 4−5), and poor quality (score <4), whereas observational studies were scored out of 10 and classified as good quality (score 6−10), fair quality (score 4−5), and poor quality (<4).

## 3 RESULTS

Of the final chosen 31 studies, 27 (87.10%) were case reports and series, and 4 (12.90%) were identified as cohort studies.

### 3.1 Demographics

In total, 111 people were found to have experienced the outbreak of PR and PR‐like presentation after receiving the vaccination for Covid‐19. They were primarily female, comprising 36 (55.38%) cases. Twenty‐nine (44.62%) patients were male, while the gender of 46 from 111 people had not been specified in the studies. The average age of incidence was calculated to be 44.92 years. The age of 32 participants was not recorded. Of 101 cases with a specified number of vaccination doses administered before presentation, 63 (62.37%) comprised the first dose, and 38 (37.62%) accounted for the onset after the second dose. Studies included the manifestation of different kinds of vaccines manufactured by different companies. People predominantly reported to have received the mRNA vaccine; of Pfizer; BioNTech BNT162b2, in 38 cases (35.51%), and of Moderna; mRNA‐1273 in 27 patients (25.23%) before exhibiting the symptoms of the cutaneous reaction. Two (1.86%) other mRNA vaccines were found, but the pharmaceutical company was not identified. Other vaccines included manufacturers Sinovac; COVID‐19 Vaccine (Vero Cell), Inactivated/CoronaVac, (*n* = 23, 21.49%), AstraZeneca; AZD1222 Vaxzevria, (*n* = 11, 10.28%), Bharat Biotech; SARS‐CoV‐2 Vaccine, Inactivated (Vero Cell)/COVAXIN (*n* = 3, 2.80%), Sinopharm; SARS‐CoV‐2 Vaccine (Vero Cell), Inactivated (lnCoV) (*n* = 2, 1.86%), Johnson and Johnson; Ad26.COV2.S (*n* = 1, 0.93%), while 4 of them were unknown (Table 1).

**Table 1:** Demographics, incidence, prognostic, and etiological factors of patients with pityriasis rosea and pityriasis rosea lesions.

Author names	Sample (*n*)	Mean age (years)	Gender (F = female/M = male)	Vaccine	Dose	Time from vaccination (days)	Treatment	Days to recover (weeks)
Temiz et al.^14^	31	44.9	18F & 13M	14 Pfizer &17 Coronavac	19 first & 12 second	12.7	Topical corticosteroid and antihistamine	7.8
Cyrenne et al.^15^	1	20	F	Moderna	First	2	Topical corticosteroid therapy	2
	1	40	M	Moderna	Second	N/A	Doxycycline and bilastine	3
Shin et al.^16^	1	29	M	Moderna	Second	0.083	N/A	N/A
Cohen et al.^17^	1	66	M	Pfizer	First	7	Triamcinolone 0.1% ointment	4
Marcantonio‐Santa Cruz et al.^18^	1	22	F	Pfizer	Second	2	N/A	N/A
	1	54	F	Pfizer	First	7	Topical steroids	3
Abdullah et al.^19^	1	40	M	mRNA	Second	7	0.1% Triamcinolone cream for pruritus	3
Bostan et al.^20^	1	34	F	mRNA	Second	15	Symptomatic treatment	N/A
McMahon et al.^21^	8	61.5	N/A	Moderna (38%), Pfizer (50%), Oxford‐AstraZeneca (12%)	First	13	N/A	N/A
Gökçek et al.^22^	1	68	M	Coronavac	First	10	N/A	N/A
Wang et al.^23^	1	40	M	Moderna	First	7	Prednisolone 30 mg/day for 5 days and tapered	2
Vázquez et al.^24^	1	35	M	Pfizer	First	N/A	Antihistamines and topical betamethasone	2
Adya et al.^25^	1	21	F	AstraZeneca	First	4	N/A	N/A
Mehta et al.^26^	1	24	M	AstraZeneca	N/A	1	Topical mometasone cream	N/A
Yao et al.^27^	1	19	M	Sinopharm	First	2	Valaciclovir and mometasone furoate	1
	1	51	M	Sinopharm	Second	7	Ganciclovir 250 mg bid orally	N/A
Akdaş et al.^28^	1	45	F	Coronavac	First	4	Oral antihistamine and a mid‐potency topical corticosteroid cream	3
Dormann et al.^29^	1	19	F	AstraZeneca	First	4	No treatment	N/A
Freeman et al.^30^	16	N/A	N/A	Moderna	10 first & 6 second	N/A	N/A	N/A
	8	N/A	N/A	Pfizer	3 first & 5 second	N/A	N/A	N/A
	2	N/A	N/A	AstraZeneca	First	N/A	N/A	N/A
	1	N/A	N/A	Johnson & Johnson	N/A	N/A	N/A	N/A
	2	N/A	N/A	Coronavac	1 first & 1 second	N/A	N/A	N/A
	1	N/A	N/A	N/A	First	N/A	N/A	N/A
Burlando et al.^31^	1	31	M	Pfizer	Second	30	N/A	N/A
Grieco et al.^32^	3	47	N/A	N/A	First	14	N/A	N/A
Agarwal et al.^33^	1	30	M	Covaxine	Second	3	N/A	N/A
	1	58	F	Covaxine	Second	30	N/A	N/A
	1	50	F	Covaxine	First	2	N/A	N/A
Larson MD et al.^34^	1	29	F	Moderna	First	7	Topical steroids	N/A
Ponis et al.^35^	1	42	F	Pfizer	Second	4	N/A	N/A
	1	64	M	Pfizer	First	5	N/A	N/A
Menzinger et al.^36^	1	36	F	Pfizer	Second	15	N/A	N/A
Valk et al.^37^	1	30	F	Pfizer	Second	3	Triamcinolone cream 0.1%	N/A
Rerknimitr et al.^38^	1	N/A	N/A	Coronavac	First	1	N/A	N/A
	1	N/A	N/A	Coronavac	Second	1	N/A	N/A
Buckley et al.^39^	1	23	F	Pfizer	First	7	Triamcinolone cream	N/A
Das et al.^40^	3	36.7	N/A	AstraZeneca	N/A	N/A	N/A	N/A
Niebel et al.^41^	1	63	M	AstraZeneca	First	21	Topical corticosteroids	N/A
Martora et al.^42^	1	46	F	Moderna	First	6	N/A	N/A
	1	49	M	Moderna	First	7	N/A	N/A
	1	24	F	Moderna	First	11	Topical steroids and oral histamines	N/A
Lim et al.^43^	1	24	F	AstraZeneca	First	3	Cetirizine, betamethasone valerate	2
Khattab et al.^44^	1	49	F	Pfizer	First	8	Oral histamines and topical betamethasone	4
	1	53	M	Pfizer	Second	7	N/A	N/A

### 3.2 Incidence, treatment, and recovery

The mean time of onset of PR and PR‐like eruption mentioned in the 92 cases after getting vaccinated was 8.58 days, and the meantime they took to recovery in 42 patients after the appropriate treatment was 6.44 weeks. Fifty‐one individual case studies notified the treatment prescribed to them, and most of them took steroids for recovery, 46 (90.19%) patients. Thirty‐seven (72.54%) people were given antihistamines. Other treatments advised were antibiotics (1.96%) and antivirals (3.92%), namely Doxycycline and Valaciclovir or Ganciclovir. These findings are portrayed in Table 1.

### 3.3 Morphological and histological features

Thirty‐nine participants failed to report the morphological features. Six (8.33%) out of the remaining 72 people specifically presented as atypical PR with vesicular lesions in 5 and herald patches in only 3 of them. The appearance of the rash of 34 (37.22%) out of 72 people surfaced like a typical “herald patch.” In 36 cases (50.00%), lesions presented as “Christmas tree appearance,” 8 (10.53%) patients were also found to have developed collarette scales. In addition, 26 studies reported histological findings, of which common were parakeratosis (26.92%), spongiosis (59.25%), extravasated red blood cells (23.07%), and perivascular lymphocytic infiltrates (26.92%). These principal characteristics of each case were synthesized and summarized in Table 2.

**Table 2:** Distinct characteristics of patients with pityriasis rosea and pityriasis rosea‐like lesions.

Author names	Sample (*n*)	Morphology	Distribution	Histological features	Clinical features
Temiz et al.^14^	31	26 HP followed by CTP (typical)	N/A	N/A	N/A
		5 purpuric and vesicular (atypical)	N/A	N/A	N/A
Cyrenne et al.^15^	1	Small, oval, pink to tan color thin plaques with peripheral scale, hyperpigmented center, and trailing scale	Trunk and proximal extremities	PK, scattered dyskeratotic keratinocytes, minimal acanthosis and SG, melanin incontinence, perivascular PLI, and EV‐RBC	N/A
	1	A HP (left lateral axilla), as well as smaller plaques with peripheral scale	Left axilla, trunk, and proximal extremities	N/A	Asymptomatic
Shin et al.^16^	1	HP (right chest), multiple ovals, salmon‐colored patches	Right chest and extremities	Focal PK, SG, and superficial PLI	Asymptomatic
Cohen et al.^17^	1	Red‐brown eczematous plaque (3 cm) on the right flank and multiple two‐toned papules with a fine overlying scale	Right flank (abdomen)	Superficial PLI, EV‐RBC, nonspecific chronic dermatitis, SG, focal hyperkeratosis, and PK	N/A
Marcantonio‐Santa Cruz et al.^18^	1	Multiple ovals (0.4–2.5 sm), pink erythematous plaques with an inner collarette of scaling	The trunk and proximal extremities and following the lines of cleavage	Mild psoriasiform hyperplasia, focal PK and SG, a superficial PLI with scattered eosinophils and focal EV‐RBC	Itchy, asymptomatic
	1	Multiple small scaly oval plaques, a more extensive plaque	Trunk left arm	N/A	Asymptomatic
Abdullah et al.^19^	1	A single larger erythematous patch with the scale (back)with smaller lesions	Trunk, back, and proximal extremities (the arms, thighs, chest, abdomen, and flanks in a Blaschkoid distribution with no mucosal or acral involvement)	N/A	Asymptomatic
Bostan et al.^20^	1	Multiple, tan‐colored, annular, thin plaques with central clearing and peripheral scales, HP followed by smaller patches	Proximal extremities (flexor aspects of arms and lateral thighs)	N/A	Asymptomatic
McMahon et al.^21^	8	Oval, pink edematous papules and plaques, with central crust and with trailing scale	Trunk, extremities	SG, interface changes, and dermal eosinophils	N/A
Gökçek et al.^22^	1	Erythematous, collarette scaly plaques (CTP), also HP followed by scaly oval patches	Trunk, back, abdomen, proximal extremities	N/A	Itchy, asymptomatic
Wang et al.^23^	1	Multiple variously sized oval erythematous papules and plaques with central darkening and collarette scales (CTP)	Lower abdomen initially, but spread to the neck, trunk, and four limbs afterward (along the cleavage lines)	PK with slight acanthosis and mild SG, PLI, and eosinophilic infiltrate in the superficial dermis	Asymptomatic
Vázquez et al.^24^	1	A single oval erythematous lesion appeared and progressed insidiously to a papulosquamous rash	Thigh, trunk, proximal extremities	N/A	N/A
Adya et al.^25^	1	Discrete and coalescent papulovesicular lesions, red dots in an irregular distribution, central hemorrhagic crusting with collarette scaling	Trunk, back, proximal extremities	Epidermal SG, PLI in the papillary dermis, and EV‐RBC in the papillary and reticular dermis	mild febrile episode 2 days before the rash associated with myalgia
Mehta et al.^26^	1	Round to oval salmon‐colored papules and plaques (3 cm covered by fine white scales	Trunk, back, axilla	N/A	Asymptomatic
Yao et al.^27^	1	Pruritus papulosquamous, several oval pink‐to‐brown‐colored thin scaly plaques	Trunk and proximal extremities	N/A	N/A
	1	Itchy fusiform patches, annular and oval lesions covered by thin scales across a CTP	Neck, trunk, bilateral groins, & proximal extremities	N/A	Itchy, asymptomatic
Akdaş et al.^28^	1	Oval plaques (2 cm) with a peripheral collarette scaling consistent with the HP (right scapula and right breast) CTP	Right scapula, right breast, trunk, proximal extremities, along the cleavage lines	Focal PK in mounds with exocytosis of lymphocytes, epidermal SG, and EV‐RBC in the dermis	Asymptomatic
Dormann et al.^29^	1	Generalized erythematous plaques (2−40 mm) with scaly collarettes	Trunk, proximal extremities	N/A	Asymptomatic
Freeman et al.^30^	16	N/A	N/A	N/A	N/A
	8	N/A	N/A	N/A	N/A
	2	N/A	N/A	N/A	N/A
	1	N/A	N/A	N/A	N/A
	2	N/A	N/A	N/A	N/A
	1	N/A	N/A	N/A	N/A
Burlando et al.^31^	1	Diffuse, only mildly pruritic	N/A	N/A	Headache, asthenia‐associated symptoms
Grieco et al.^32^	3	N/A	N/A	N/A	N/A
Agarwal et al.^33^	1	Multiple oval‐shaped erythematous scaly plaques with HP	N/A	N/A	N/A
	1	N/A	N/A	N/A	N/A
	1	N/A	N/A	N/A	N/A
Larson et al.^34^	1	Small papules and patches with a collarette of scale	Chest, abdomen, and back	Mild epidermal hyperplasia with SG and mounds of PK	N/A
Ponis et al.^35^	1	N/A	Neck, upper limb, trunk	N/A	N/A
	1	Erythematous rash	Trunk	N/A	N/A
Menzinger et al.^36^	1	2 cm, thin and scaly plaque. Erythematous papules of varying sizes	Trunk and upper extremities, distributed along the cleavage lines	N/A	N/A
Valk et al.^37^	1	N/A	N/A	N/A	N/A
Rerknimitr et al.^38^	1	N/A	N/A	N/A	N/A
	1	N/A	N/A	N/A	N/A
Buckley et al.^39^	1	Numerous oval to annular salmon‐colored plaques with a collarette of scale. HP (right inferior breast)	Trunk and breast	N/A	N/A
Das et al.^40^	3	Papulosquamous lesions in an inverted CTD	Trunk	N/A	N/A
Niebel et al.^41^	1	Non‐pruritic pale erythematous exanthema. Singular lesions (5 cm), CTP	Trunk	A superficial PLI with interface dermatitis and EV‐RBC	N/A
Martora et al.^42^	1	Erythematous and desquamative lesions	Trunk and legs	N/A	N/A
	1	Erythematous and desquamative lesions	Trunk	N/A	N/A
	1	Erythematous and desquamative lesions	Trunk	N/A	N/A
Lim et al.^43^	1	N/A	N/A	N/A	N/A
Khattab et al.^44^	1	Macular rash on the trunk and oval scaly HP on the abdomen	Trunk, proximal extremities, and abdomen	N/A	Itch
	1	Macular rash with HP, annular plaque	Upper trunk and abdomen	N/A	Itch

Abbreviations: CTP, christmas tree pattern; EV‐RBC, extravasated red blood cells; HP, herald patch; PK, parakeratosis; PLI, perivascular lymphocytic infiltrates; SG, spongiosis.

### 3.4 Distribution and associated symptoms

The distribution of the rashes was calculated to be most common in the trunk, 28 cases (87.5%) of the 32 cases with this data, which extended to proximal extremities (*n* = 13, 40.62%), back (*n* = 5, 15.62%), and abdomen (*n* = 7, 21.87%). Only a few associated symptoms were noticed, which included itch (*n* = 5, 31.25%), fever (*n* = 1, 6.25%), myalgia (*n* = 1, 6.25%), asthenia (*n* = 1, 6.25%), headache (*n* = 1, 6.25%). Twelve out of 16 people did not experience symptoms (75%). Table 2 outlines these features.

## 4 DISCUSSION

To our best understanding and knowledge, this is the first comprehensive study to systematically review the manifestation of PR and PR‐like eruptions after the Covid‐19 vaccination. It covers aspects like its morphological, histological, and clinical features and discusses the time of onset from the dose of vaccination and the time taken to recover after appropriate treatment options.

SARS‐CoV‐2 has spike proteins that pierce and infect host cells, inducing cytokine‐storm hence the inflammatory reaction causing PR/PR‐LE after the infection is common and has been observed in studies. Like the vaccines of Pfizer and Moderna in our study, the mRNA vaccines use the virus's genetic code to make copies of those spike proteins as a modified RNA sequence with lipid nano protein encapsulating it and eliciting a similar reaction to the virus.^45^ Due to exposure to the virus antigen, the cell‐mediated immune response increases T cells and cytokines.^46^ This immune dysregulation can lead to inflammation and reactivation of latent viral infections, like human herpesvirus‐ HHV6 and HHV7, which are known to have been associated with PR.^31^ The herpesvirus settles in the salivary glands and is usually transferred in the first 2 years of life. Late presentation of PR and PR‐LE can explain this etiology.^47^ A study shows the manifestation of PR during the pandemic increased approximately five times as compared to the same time last year.^48^ This also shows that it is possible for people with an immunocompromised state to develop this lesion, as similarly reported in a case study by Maria Cristina Pedrazini et al. Other Covid‐19 vaccines that were reported to have a similar effect in this study were Adenovirus vectored (AstraZeneca, Johnson & Johnson) and Inactivated (Sinopharm, CoronaVac Sinovac, Covaxine). According to WHO, the general covid‐19 vaccine administration pattern was dominated by mRNA vaccines. This could be the reason why most of our patients presented after doses of Pfizer or Moderna.^49^ This dermatological manifestation has also been witnessed after vaccination against other infections like tuberculosis, smallpox, diphtheria, influenza, diphtheria‐pertussis‐tetanus, influenza A, tetanus, pneumococcus, papillomaviruses, hepatitis B, and yellow fever, after a range of 5−17 days of the administration, unlike in our study in which time of onset range after Covid‐19 vaccines was 24 h to 30 days.^18^

The acute cutaneous viral reaction commonly occurs in teenagers and young adults (10−35 years). This may be because they have a more active immune response as compared to the other age groups. Peripheral dendritic cells, along with CD4+, CD8+, and T‐cells, linearly decrease as we grow older. The pediatric population has been found to have a noticeable increase in cytokines, especially IL‐6, IFN‐γ, and IL‐10. This can also help justify their severe immune response to the covid‐19 infection.^50^ Similarly, prevalence is slightly higher in females, due to their stronger cellular and humoral immune responses than males, possibly because of the sex hormones; estrogen, prolactin, progesterone, and androgens, which play an important role in immunomodulation and lead to inflammatory and autoimmune diseases.^51^, ^52^ Other studies have reported a high incidence of PR/PR‐LE in pregnant females, possibly due to altered immune function. It reported that 57% of 61 pregnant women developed PR within 15 weeks of gestation, and 13% of them suffered from spontaneous abortion.^47^ Pregnancy can be pro or anti‐inflammatory, depending on the gestation time. The first trimester, which is the proinflammatory phase, can explain the possible reason for the development of this disease during the first trimester.^53^

Significant triggers other than autoimmunity for PR can be bacterial, viral, environmental factors like seasonal change, atopy, stress, or drugs such as ACE inhibitors, like captopril, Allopurinol, hydrochlorothiazide, barbiturates, nimesulide, and metronidazole.^20^, ^22^ The drug‐induced reaction is not commonly associated with HHV6/HHV7 reactivation. It mostly does not present with prodromal symptoms, herald patch, or eosinophilia under a microscope.^18^ It might resolve after withdrawal of the drug.^54^

In this review, cases displayed and summarized a wide set of morphological features, typically appearing as a herald patch localized in the trunk initially, then spreading to other areas to mimic a pine tree. Atypical PR has unusual characteristics like some cases displayed vesicular or purpuric large but fewer patches in number as a coalescent, with an unconventional distribution like in the axilla, groin, limbs, and rarely face and neck, with trunk spared.^54^, ^55^ PR‐LE is distinguished from PR by a lack of prodromal symptoms and heraldic patches. Its common attributes include diffuse and itchy rash with dermal eosinophils and interface dermatitis histologically. On the contrary, a classic PR case shows spongiosis, lymphocyte infiltration, extravasated blood cells, and parakeratosis.^19^, ^56^

A study shows that 75% of people with herald patch present with symptoms like headache, insomnia, irritability, and mostly fatigue, but our review was not suggestive of this. The patients were mainly asymptomatic, and symptoms reported in our studies were itch, fever, myalgia, asthenia, and headache.^57^

Even though PR is a self‐limiting reaction, drugs for symptomatic treatment and relief were prescribed, like mometasone furoate for the itch.^27^ Corticosteroid, due to their anti‐inflammatory properties, was commonly prescribed in our included cases, namely triamcinolone, prednisone, and betamethasone. They are also known to have fewer potential side effects.^47^ Similarly, antibiotics were used because they have anti‐inflammatory and immunomodulatory effects. Acyclovir, advised in our two case reports, is an antiviral drug that inhibits DNA synthesis in the herpesvirus. Due to the positive association of HHV‐6 and HHV‐7, it may be effective in resolving rashes of PR and PR‐LE.^27^, ^58^ An alternative treatment suggested in other studies is l‐lysine, taken 3 g in the first 3 days, then 500 m for the next 30 days as a maintenance dose. l‐Lysine targets the intestines and kidneys to increase l‐Arginine excretion, interfering in protein synthesis and viral replication.^59^ It usually takes around 6−8 weeks for the lesions to resolve but can take as long as 3−6 months. The average time calculated to recover in a few cases of our research after being treated with appropriate drugs was less, indicating the treatment's effectiveness to treat promptly.^12^ Low‐dose ultraviolet A1 phototherapy has been proven to produce satisfactory results for the severe presentation of PR, with only mild side effects reported; pruritus, erythema, polymorphous light eruption, and a burning sensation, that resolved within 1 month of completing the required course. It may be more favorable in the early stages as it leads to apoptosis of T‐cells along with regulation of proinflammatory cytokines and there is less inflammatory infiltrate in the late stages.^60^

Reactivation of PR was reported in one of our cases with the follow‐up data. It was presented after 4 days of administering the second dose of the CoronaVac vaccine. The lesions were similar, but they were self‐resolved, and the duration was shorter than the first occurrence, 1 week.^28^ A retrospective cohort study supports this and states the relapse rate to be 3.7%. Even though the etiology for this is not known, it is hypothesized to be related to the reactivation of HHV6 and 7, with factors like stressful situations acting as a stimulus. Another study also describes recurrences of PR/PR‐LE, with varying presentation and severity.^61^, ^62^

### 4.1 Limitations

Our systematic review has a few limitations. Many participant studies had information bias, as many key study variables were missing from existing reports, affecting the quality of our review. Biopsies were not performed in some cases, which led to inadequate histological knowledge. There was a lack of follow‐up in a few records, because of which we could not determine the actual effectiveness of the treatment prescribed. Since the studies chosen for data extraction reported cases from different races and backgrounds, there were variations in characteristics reported of PR and PR‐LE; for example, the findings were presented according to each patient's skin color and texture, which is why one specific diagnostic criterion could not be used. Despite the vigorous screening process, our review was limited in generalizability, as the data available in the selected studies and the sample size was small and could only be applied to a narrow population.

## 5 CONCLUSIONS

In conclusion, this review summarized the manifestation and characteristics of PR/PR‐LE reported in a significant number of people after the administration of the Covid‐19 vaccination, hence establishing a positive association. Our study supports the reaction to be mild, presenting as mostly asymptomatic (75%) or with minor symptoms (25%). Its prompt treatment has also reported safe and effective treatment options, including corticosteroids and acyclovir. This can help improve knowledge and perception regarding such side effects of the Covid‐19 vaccine and avert people from discouraging vaccination. Further in‐depth studies and clinical trials should be conducted to confirm the association further and fully understand the path‐etiology of the reaction, which can also allow them to improve their vaccine‐making strategies.

## AUTHOR CONTRIBUTIONS

Iman Khan and Ahmed A. Elsanousi conceptualized the study and did formal analysis. Amena M. Shareef and Sameer S. Tebha contributed in methodology and project administration. Aabiya Arif and Sana Gul took part in investigation and software. All authors equally participated in writing of the original draft, reviewing, and editing it. All authors have read and approved the final manuscript.

## CONFLICT OF INTEREST STATEMENT

The authors declare no conflict of interest.

## Supporting information

Supporting information.

## Data Availability

All data are present in the main manuscript and supplemental file.
