# Supplementary material for: Manifestation of pityriasis rosea and pityriasis rosea‐like eruptions after Covid‐19 vaccine: A systematic review
Source: Immun Inflamm Dis. 2023 Apr 12;11(4):e804. doi: 10.1002/iid3.804 (PMC10091373; doi:10.1002/iid3.804)
Supplement: Supplementary file 1 — Supporting information. [file IID3-11-e804-s001.docx]

Table S1: Search Strategy

| Strategy | Databases= results |
| --- | --- |
| - (Covid-19 OR COVID-19 Virus Disease OR COVID-19 Virus Infection OR SARS-CoV-2 infection OR 2019 Novel Coronavirus Disease OR Coronavirus Disease-19 OR Severe Acute Respiratory Syndrome Coronavirus 2 Infection OR COVID-19 OR COVID19 Virus Vaccines OR SARS-CoV-2 Vaccine OR Coronavirus Disease 2019 Vaccines OR COVID-19 Vaccine OR Novel Coronavirus Vaccines OR 2019 nCoV Vaccine mRNA 1273 OR Moderna COVID-19 Vaccine OR Elasomeran OR Moderna COVID-19 Vaccine RNA OR mRNA-1273 OR TAK-919 OR mRNA-1273.211 OR Ad26.COV2.S OR Johnson and Johnson Covid-19 Vaccine OR JNJ-78436735 OR Vaccine, BNT162 OR COVID-19 Vaccine Pfizer-BioNTech OR Pidacmeran OR BNT-162C2 OR Abdavomeran OR BNT-162B1 OR BNT-162A1 OR Tozinameran OR Comirnaty OR BNT-162B2 OR ChAdOx1 nCoV 19 OR Oxford-AstraZeneca COVID-19 Vaccine OR ChAdOx1 COVID-19 Vaccine OR Covishield OR AZD-1222 OR Sinopharm OR Sinovac OR Coronavac ) AND (Manifestation, Skin OR Manifestations, Skin OR Skin manifestation OR Cutaneous reaction OR Cutaneous reactions OR Cutaneous manifestation OR Pityriasis Rosea OR Papulosquamous OR maculopapular lesions OR Pityriasis Rosea-like OR Pityriasis Rosea OR human herpesvirus 6-7 OR Herald patch OR Christmas pattern OR scaly patches) | Pubmed= 1,157  Directory of Open Access Journals =0  MedRxiv=0  BioRxiv=0 |
| - (Covid-19 Virus vaccine OR SARS-COV-2 vaccine OR corona virus vaccine) AND (Pityriasis Rosea OR Papulosquamous OR maculopapular lesions) | Sciencedirect=188  Google scholar 265  Directory of Open Access Journals =0  MedRxiv=0  BioRxiv=0 |
| - (Covid-19 Virus vaccine OR SARS-COV-2 vaccine OR corona virus vaccine OR Pfizer OR Moderna OR astrazeneca OR Sinopharm OR Sinovac OR corona vac OR johnson and johnson OR sino shield) AND (Pityriasis Rosea OR Papulosquamous OR maculopapular lesions) | Cochrane = 13  Directory of Open  Access Journals =0  MedRxiv=0  BioRxiv=0 |
| - Covid19 Vaccine AND Pityriasis Rosea | Directory of Open Access Journals =6  MedRxiv=0  BioRxiv=0 |

Table S2: Case Study and Series:

| # | First  author | Was the study question or objective clearly stated? | Was the study population clearly and fully described, including a case definition? | Were the cases consecutive? | Were the subjects comparable? | Was the intervention clearly described? | Were the outcome measures clearly defined, valid, reliable, and implemented consistently across all study participants? | Was the length of follow-up adequate? | Were the statistical methods well described? | Were the results well described? | Total | Level of Evidence |
| --- | --- | --- | --- | --- | --- | --- | --- | --- | --- | --- | --- | --- |
| 1 | Selami Aykut Temiz Et al. | yes | yes | no | yes | yes | Yes | Yes | N/A | N/A | 6 | good |
| 2 | B.M. Cyrenne Et al. | yes | Yes | yes | N/A | Yes | Yes | Yes | NA | N/A | 6 | good |
| 3 | Sun Hye Shin Et al. | yes | Yes | N/A | N/A | Yes | Yes | no | N/A | N/A | 4 | fair |
| 4 | Olivia G. Cohen Et al. | Yes | Yes | N/A | N/A | yes | yes | yes | N/A | N/A | 5 | fair |
| 5 | O.Y. Marcantonio-Santa Cruz Et al | Yes | Yes | no | N/A | Yes | Yes | Yes | N/A | N/A | 5 | fair |
| 6 | Lina Abdullah Et al. | Yes | Yes | N/A | N/A | Yes | Yes | Yes | N/A | N/A | 5 | fair |
| 7 | Ecem Bostan Et al | Yes | Yes | N/A | N/A | Yes | Yes | No | N/A | N/A | 4 | fair |
| 8 | Gözde Emel Gökçek Et al. | Yes | Yes | N/A | N/A | Yes | Yes | No | N/A | N/A | 4 | fair |
| 9 | Wang C Et al | Yes | Yes | N/A | N/A | Yes | Yes | Yes | N/A | N/A | 5 | Fair |
| 10 | A M Carballido Vázquez Eet al. Magoon et al. | Yes | Yes | N/A | N/A | Yes | Yes | Yes | N/A | N/A | 5 | Fair |
| 11 | Keshavmurthy A. Adya Eet al. | Yes | Yes | N/A | N/A | Yes | Yes | No | N/A | N/A | 4 | Fair |
| 12 | H. Mehta Eet al. | Yes | Yes | No | No | Yes | Yes | No | N/A | N/A | 4 | Fair |
| 13 | L. Huang,Z. Yao Et al. | Yes | Yes | Yes | Yes | Yes | Yes | Yes | NA | N/A | 7 | Good |
| 14 | E. Akdaş Et al. | Yes | Yes | No | No | yes | Yes | yes | N/A | N/A | 5 | Fair |
| 15 | Harald Dormann Et al. | Yes | Yes | N/A | N/A | Yes | Yes | No | N/A | N/A | 4 | Fair |
| 16 | Martina Burlando Et al. | Yes | Yes | N/A | N/A | Yes | Yes | No | N/A | N/A | 4 | Fair |
| 17 | Agarwal A1 Et al. | Yes | yes | Yes | Yes | yes | No | no | N/A | N/A | 5 | fair |
| 18 | Valerie Larson MD Et al. | Yes | Yes | N/A | Yes | Yes | No | No | N/A | N/A | 4 | Fair |
| 19 | E. Farinazzo,G. Ponis Et al | Yes | Yes | yes | Yes | Yes | No | No | N/A | N/A | 5 | fair |
| 20 | M. Tihy,S. Menzinger Et al. | Yes | yes | N/A | N/A | yes | Yes | No | N/A | N/A | 4` | Fair |
| 21 | Brittany Valk Et al. | Yes | Yes | N/A | Yes | Yes | No | No | N/A | N/A | 4 | fair |
| 22 | Jordan E Buckley et al. | yes | yes | N/A | N/A | yes | Yes | No | N/A | N/A | 4 | Fair |
| 23 | P. Das et al. | yes | yes | no | Yes | yes | No | No | N/A | N/A | 4 | fair |
| 24 | Dennis Niebel et al | yes | yes | no | no | yes | yes | no | N/A | N/A | 4 | fair |
| 25 | Fabrizio Martora et al | yes | yes | yes | Yes | yes | Yes | no | N/A | N/A | 6 | good |
| 26 | .N. Yu et al. | yes | yes | No | No | yes | yes | yes | N/A | N/A | 5 | fair |
| 27 | Elina Khattab et al. | yes | yes | yes | yes | yes | no | yes | N/A | N/A | 6 | good |

Table S3: Observational Cohort and Cross-Sectional Studies

| s. no | First author | Was the research question or objective in this paper clearly stated? | Was the study population clearly specified and defined? | Was the participation rate of eligible persons at least 50%? | Were all the subjects selected or recruited from the same or similar populations (including the same time period)? Were inclusion and exclusion criteria for being in the study prespecified and applied uniformly to all participants? | Was a sample size justification, power description, or variance and effect estimates provided? | For the analyses in this paper, were the exposure(s) of interest measured prior to the outcome(s) being measured? | Was the timeframe sufficient so that one could reasonably expect to see an association between exposure and outcome if it existed? | For exposures that can vary in amount or level, did the study examine different levels of the exposure as related to the outcome (e.g., categories of exposure, or exposure measured as continuous variable)? | Were the exposure measures (independent variables) clearly defined, valid, reliable, and implemented consistently across all study participants? | Was the exposure(s) assessed more than once over time? | Were the outcome measures (dependent variables) clearly defined, valid, reliable, and implemented consistently across all study participants? | Were the outcome assessors blinded to the exposure status of participants? | Was loss to follow-up after baseline 20% or less? | Were key potential confounding variables measured and adjusted statistically for their impact on the relationship between exposure(s) and outcome(s)? | total |
| --- | --- | --- | --- | --- | --- | --- | --- | --- | --- | --- | --- | --- | --- | --- | --- | --- |
| 28 | Devon E. McMahon Et al. | yes | yes | N/A | Yes | no | yes | N/A | No | yes | No | yes | N/A | No | N/A | 6 |
| 29 | Esther E.FreemanMD Et al | yes | no | N/A | Yes | no | yes | N/A | No | yes | No | yes | N/A | No | N/A | 5 |
| 30 | Teresa Grieco Et al | yes | yes | N/A | Yes | no | yes | N/A | No | yes | No | yes | N/A | No | N/A | 6 |
| 31 | P. Rerknimitr Et al. (38) | yes | no | N/A | Yes | no | yes | N/A | No | yes | No | yes | N/A | No | N/A | 5 |

|  | Level of Evidence |
| --- | --- |
| 28 | Good |
| 29 | fair |
| 30 | Good |
| 31 | fair |
